# KRAS is a prognostic biomarker associated with diagnosis and treatment in multiple cancers

**DOI:** 10.3389/fgene.2022.1024920

**Published:** 2022-10-18

**Authors:** Da Zhao, Lizhuang Wang, Zheng Chen, Lijun Zhang, Lei Xu

**Affiliations:** ^1^ Institute of Fundamental and Frontier Sciences, University of Electronic Science and Technology of China, Chengdu, China; ^2^ School of food and drug, Shenzhen Polytechnic, Shenzhen, China; ^3^ Beidahuang Industry Group General Hospital, Harbin, China; ^4^ School of Electronic and Communication Engineering, Shenzhen Polytechnic, Shenzhen, China

**Keywords:** KRAS, tumor stage, cell infiltration, functional enrichment analysis, tumor microenvironment, prognostic biomarker

## Abstract

*KRAS* encodes K-Ras proteins, which take part in the MAPK pathway. The expression level of KRAS is high in tumor patients. Our study compared *KRAS* expression levels between 33 kinds of tumor tissues. Additionally, we studied the association of *KRAS* expression levels with diagnostic and prognostic values, clinicopathological features, and tumor immunity. We established 22 immune-infiltrating cell expression datasets to calculate immune and stromal scores to evaluate the tumor microenvironment. *KRAS* genes, immune check-point genes and interacting genes were selected to construct the PPI network. We selected 79 immune checkpoint genes and interacting related genes to calculate the correlation. Based on the 33 tumor expression datasets, we conducted GSEA (genome set enrichment analysis) to show the *KRAS* and other co-expressed genes associated with cancers. *KRAS* may be a reliable prognostic biomarker in the diagnosis of cancer patients and has the potential to be included in cancer-targeted drugs.

## 1 Introduction

*Cancer* is a severe life-threatening disease that affects a large number of patients worldwide (Cao et al., 2021). Breast cancer has become the most common cancer in new possibilities, followed by prostate cancer, lung cancer, *etc.* (Wang et al., 2021). *KRAS* was first recognized in the Kirsten rat sarcoma virus, which encodes the p21 protein to induce virus transformation (Scolnick et al., 1979). The proteins of *KRAS* are located in the cell membranes, and on its C-terminus, there is an isoprene group (Welman et al., 2000). *KRAS* protein encodes the GTPase enzyme, which is a part of the MAPK pathway and acts as a switch on/off of transformation between GTP (guanosine triphosphate) and GDP molecules (Tsuchida et al., 1982). In mammalian cells, *KRAS* has two kinds of protein products, K-Ras4A and K-Ras4B, which are encoded by alternative exon4 (Welman et al., 2000). Many studies have observed that *KRAS* effectors affect the interactions of cells and their extracellular environment, which could also regulate cell growth, cell motility and cell metabolism (Tang et al., 2018; Gu et al., 2021; Hu et al., 1990; Hu et al., 2020; Yu et al., 2018).

*KRAS* is a signal transducer protein that binds to GTP in the MAPK pathway; the mutation of *KRAS* has been discovered in a quarter of human cancers (Pantsar, 2020). Based on the data in COSMIC, missense mutations of *KRAS* frequently occur in pancreatic, colorectal and lung cancers (Forbes et al., 2011; Ao et al., 2021; Li et al., 2021; Luo et al., 2021; Yu et al., 2021). Homozygous deletions of *KRAS* are the most frequent genetic alteration in pancreatic epithelial adenocarcinoma (PDAC), which results in cell metastasis and the transformation of cancer tumors (Chang et al., 2014). The proto-oncogenes are closely related to multiple cancers, such as cardio-facio-cutaneous syndrome (Niihori et al., 2006), ductal carcinoma of the pancreas (Hartman et al., 2012), leukemias (Singh et al., 2021), mucinous adenoma (Hartman et al., 2012), and noonan syndrome (Ando et al., 2021). The sequences of mutations in *KRAS* affect the function of genes, oncogenes, tumor-suppressor genes and stability genes and are the critical element for tumorigenesis (Vogelstein and Kinzler, 2004). *KRAS* mutations have been well characterized in 30%–50% of colorectal cancers (Andreyev et al., 2001). *KRAS* proteins are activated when transmembrane receptors are present, which include serine/threonine kinase, GTP enzyme activating protein (GAP), phosphatidylinositol 3-kinase (PI3K) and GEF (Shields et al., 2000). The abnormal activation of GTP binding of mutated *KRAS* protein leads to the unregulated growth of downstream cells (Arrington et al., 2012). Mutations at codon 12 and position 2 (GGT-GAT) of *KRAS* appear to be most common in colorectal cancer (Capella et al., 1991). The wild-type allele of KRAS is a suppressor in mouse lung cancer (Westcott et al., 2015).

Mutation sites of the *KRAS* gene have to be considered to be an effective way to develop new cancer treatment schemes (Hu et al., 2022a). Efforts to utilize KRAS and related genes as targets to explore drugs for cancer have been undertaken for years, and inhibitor drugs to block KARS^G12C^ have been developed (Xu et al., 2022). RAF1 could be an essential target to block KRAS mutant cancers (Drosten and Barbacid, 2020). EFGR-inhibiting drugs suppress the *KRAS* expression level in A549 lung cancer cells to inhibit cell proliferation (Zarredar et al., 2019).

Possibility*, KRAS* may be considered a genetic diagnosis potential biomarker of multiple malignant neoplastic diseases. First, we compared the survival data of patients with 33 kinds of tumors. Second, we analyzed the *KRAS* expression level with the characteristics of tumor patients, tumor stage, tumor microenvironment, and immune cell infiltration of 33 kinds of tumors; finally, we investigated the molecular mechanisms by GO and KEGG analysis. This study explores the molecular relationships between *KRAS* genes and cancer, which is crucial for developing new biomarkers and effective prevention and treatment of tumors.

## 2 Materials and methods

### 2.1 Data sets collection and process

We obtained 33 kinds of tumors sequencing datasets(10,327 tumor samples and 730 normal samples) somatic mutation and survival data from the UCSC Xena database (http://xena.ucsc.edu/). Subsequently, we used R language to convert the gene ID, extract the transcription data and analyze the differential expression of genes, analyze and draw, and the packages involved were R (version 4.2.1), BiomaRt (version 2.52.0, Functional Annotation Retrieval), dplyr (version 2.52.0, Data manipulation) and ggpubr (version 0.4.0, Data visualization).

### 2.2 Correlation analysis between *KRAS* expression and prognosis of tumor patients

In this experiment, we used existing data to investigate the survival status information from the UCSC Xena database, which contained 10,327 tumor samples and 730 normal samples. We gathered the survival status, which included disease-specific survival (DSS), disease-free interval (DFI) and progression-free interval (PFI) status data and time information for prognostic analysis. We divided the differentially expressed *KRAS* data into high-value and low-value groups. Furthermore, the prognostic value in 33 tumors was calculated by the Kaplan–Meier survival estimate method. The R packages utilized for this analysis were limma (version 3.9, Analyzing microarray and RNA-seq data), survival (version 3.3-1, Survival analysis), survminer (version 0.4.9, Drawing survival curves) and forestplot (version 2.0.1, Advanced Forest plot using ‘grid’ graphics).

### 2.3 Correlation analysis of *KRAS* gene expression and tumor stage, tumor mutation burden and microsatellite instability

Stage information containing the diagnostic and prognostic value of cancers was downloaded from the UCSC Xena database(http://xena.ucsc.edu/); approximately 8,099 tumor samples were divided into 3-4 stages. The limma and ggpubr packages of R were introduced to calculate the *KRAS* expression quantity and show the relevance between *KRAS* genes and tumor stage. Approximately 11,057 samples combined with *KRAS* expression data were used for TMB analysis by the Spearman correlation test. The fmsb(version 0.7.3, Medical and Health Data Analysis) package was used to create a correlation radar plot. We calculated the microsatellite instability (MSI) scores combined with the *KRAS* expression data by the Spearman correlation test, and a rader plot between tumors and *KRAS* genes was created. The fmsb package was used to create a correlation radar plot.

### 2.4 Correlation analysis of *KRAS* gene expression and the tumor microenvironment

The tumor microenvironment (TME) is considered to be the cells, tissues and matrix around a tumor; immune cells and stromal cells are considered diagnostic indicators of cancer development. We utilized ESTIMATE for predicting tumor purity by calculating stromal and immune cell infiltration (Yoshihara et al., 2013). Approximately 11,057 samples from 33 tumors were used to construct expression matrix data of *KRAS* genes. We calculated correlation between TME, MSI and expression data, tested by Spearman test methods.

### 2.5 Correlation analysis of *KRAS* gene expression and immune cell infiltration

We conducted CIBERSORT (https://cibersortstanfordedu/) to estimate the infiltration percentage of 22 immune cell types in tumors (Newman et al., 2015). According to the tumor expression data matrix file, we estimated the immune scores of all the tumor samples. Filtering through data from tumors, we approximate the correlation between cell infiltration levels and gene expression levels by Spearman’s correlation test.

### 2.6 Protein–protein interaction networks, correlation of *KRAS* with marker genes and analysis of gene enrichment

The *KRAS* genes interacted with other tumor-associated genes may help us understand tumorigenesis molecular mechanisms. We selected 33 *KRAS* coexpressed genes to construct protein–protein interaction (PPI) networks on the STRING database (https://www.string-db.org/) (Szklarczyk et al., 2019; Yu et al., 2020). We selected 79 immune checkpoint genes and interacting related genes to calculate the correlation. Based on the 33 tumor expression datasets, we conducted GSEA (genome set enrichment analysis) to show the *KRAS* and other coexpressed genes associated with cancers. This research utilized the KEGG database (https://www.kegg.jp/) and the GO database to enrich the genes (FDR <0.5).

## 3 Results

### 3.1 Transcriptional data of *KRAS* in 33 pancancer

**FIGURE 1:**
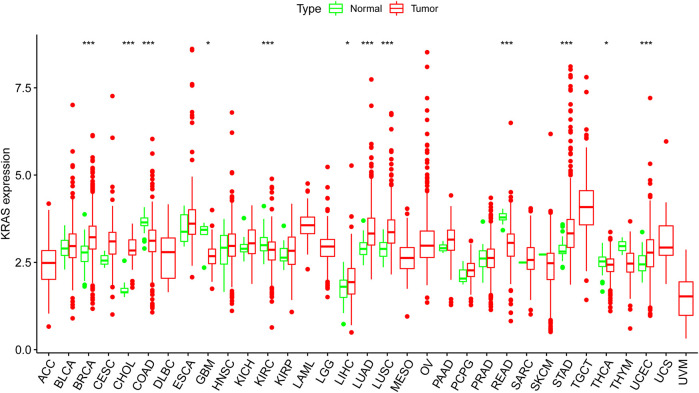
The *KRAS* expression level in cancer patients. Thirty-three kinds of tumor cancers. Expression of *KRAS* in tumors were performed by ggpubr (version 4.0.4) package of R (***p < 0.001, **p < 0.01, *p < 0.05).

We obtained the transcription datasets, somatic mutations of 33 cancer tumors from TCGA, and the survival data from the UCSC Xena database. The transcription data contained 11,057 samples which included tumor samples and normal samples. When we compared the differences between normal and tumor tissues, in most tumor tissues the expression levels of *KRAS* were higher than normal tissues. A total of 12 kinds of tumor tissues has significant difference in *KRAS* expression levels, of which 9 tumor tissues BRCA (Breast invasive carcinoma), CHOL (Cholangiocarcinoma), COAD (Colon adenocarcinoma), KIRC (Kidney renal clear cell carcinoma), LUAD (Lung adenocarcinoma), LUSC (Lung squamous cell carcinoma), READ (Rectum adenocarcinoma), STAD (Stomach adenocarcinoma), and UCEC (Uterine Corpus Endometrial Carcinoma) had an extremely significant difference; meanwhile, 3 tumor tissues (GBM (Glioblastoma multiforme), LIHC(Liver hepatocellular carcinoma), and THCA(Thyroid carcinoma) had a significant difference (Figure 1). These results indicated that cancer could lead to the abnormal expression of *KRAS*.

### 3.2 The prognostic value of the *KRAS* gene across cancers

**FIGURE 2:**
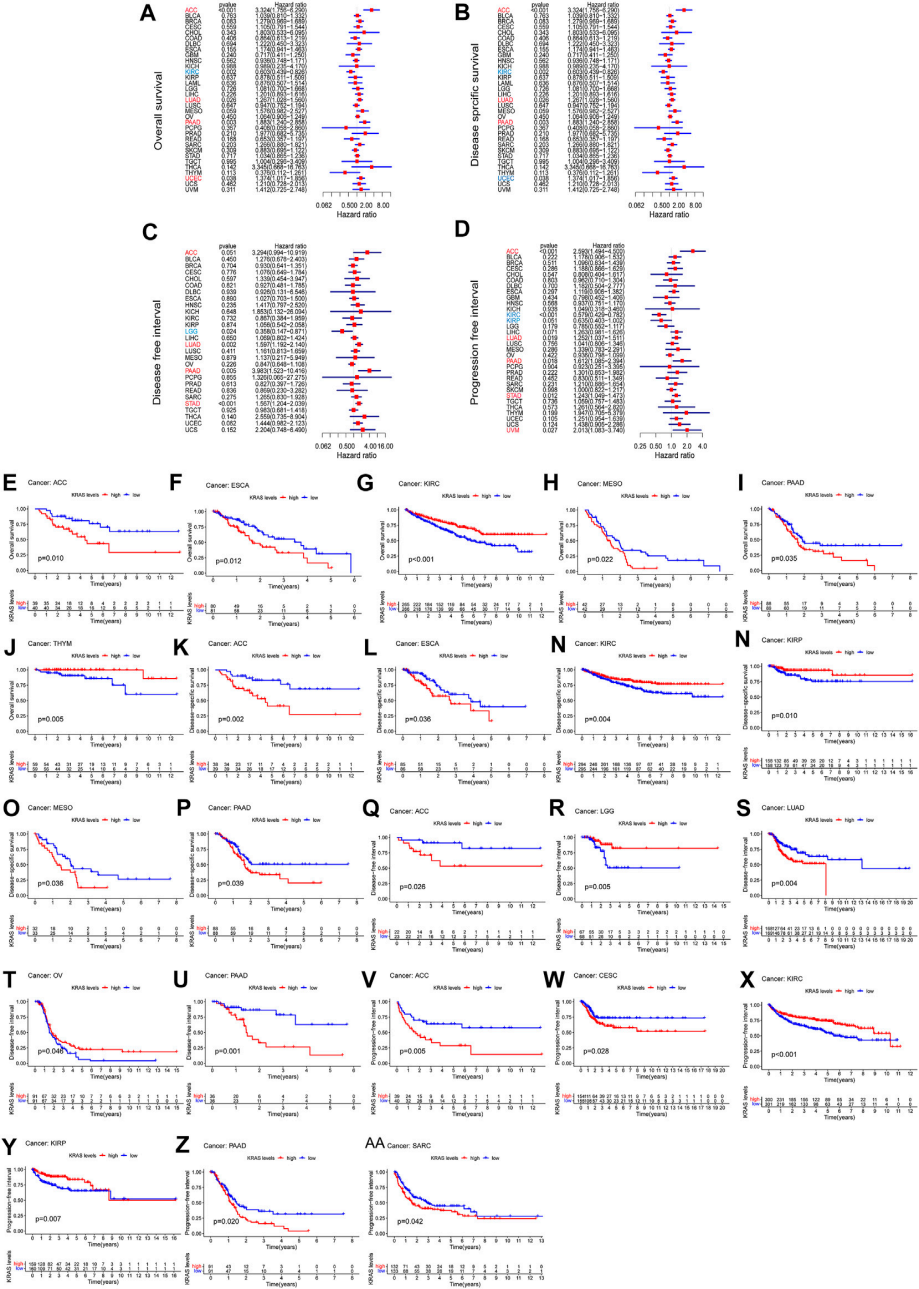
The relationship between the expression of *KRAS* and the prognosis of patients with 33 kinds of cancers in the TCGA database. **(A,E–J)** OS (overall survival) **(B,K–P)** DSS (disease-specific survival) **(C,Q–U)** DFI (disease-free interval) **(D,V–AA)** PFI (progression-free interval).

We analyzed the prognostic value of each tumor sample according to the *KRAS* expression levels with different tumors. High *KRAS* expression levels were associated with overall survival (OS) in ACC, LUAD, PAAD and UCEC (Figure 2A); poor disease-specific survival (DSS) in ACC, LUAD and PAAD (Figure 2B); poor disease-free interval (DFI) in ACC, LUAD, PAAD and STAD (Figure 2C); and poor progression-free interval (PFI) in adrenocortical carcinoma (ACC), LUAD, PAAD, STAD and uveal melanoma (UVM) (Figure 2D).

**FIGURE 3:**
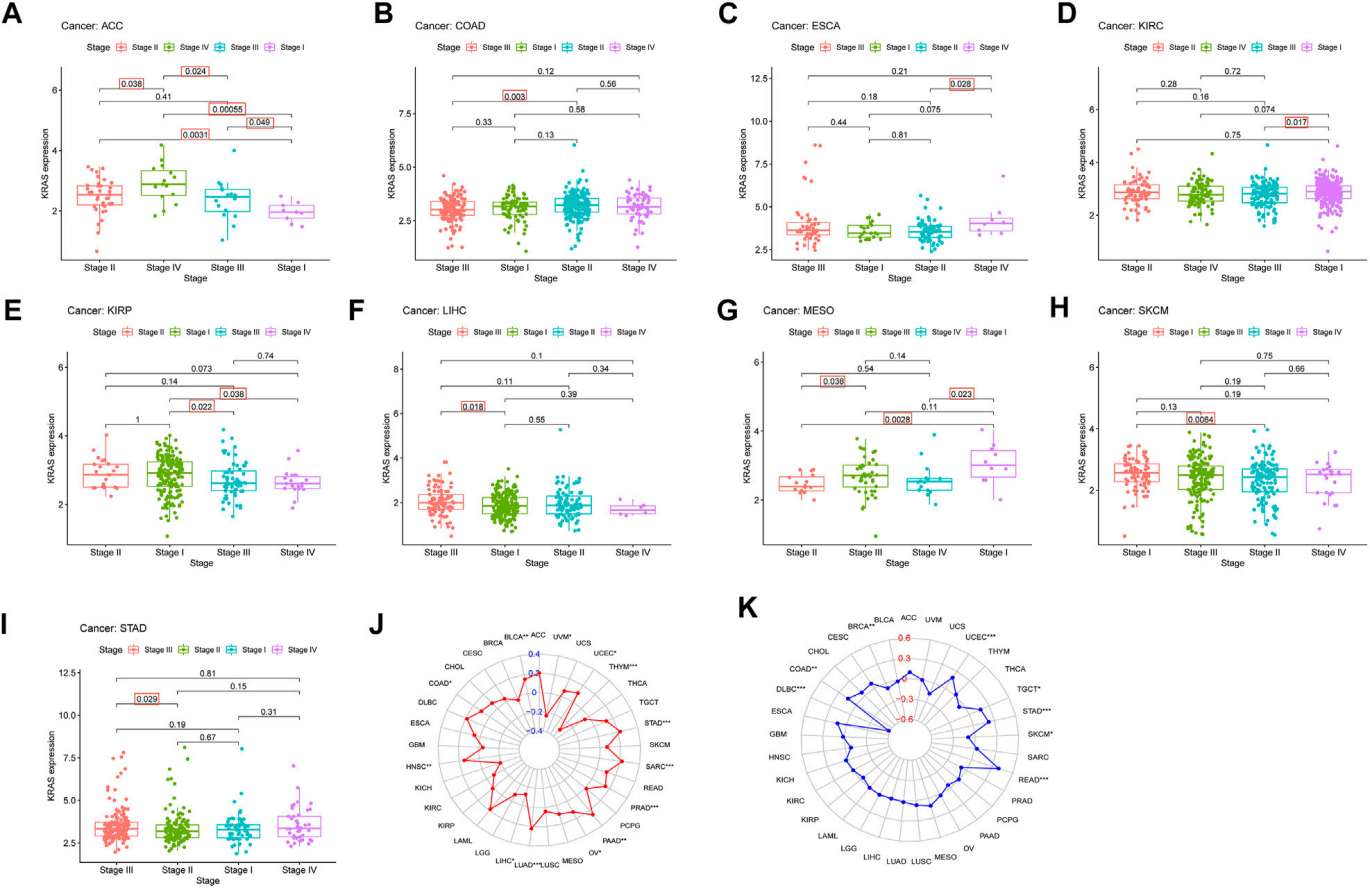
The relationship between *KRAS* expression level and pathological characteristics of tumor patients. (tumor mutation burden, microsatellite instabilitiy). **(A–I)** Relationship of *KRAS* expression level and stage grade. **(J)** Tumor mutation burden (TMB). **(K)** Microsatellite instability (MSI). *p < 0.05. **p < 0.01, ***p < 0.001.

To evaluate how effective *KRAS* is in single cancer, we calculated *p* values to analyze the association of clinical data with single cancers. High *KRAS* expression levels correlated with OS in ACC, ESCA, KIRC, PAAD and THYM of 0.01, 0.01, 0.001, 0.022, 0.035 and 0.005, respectively (Figures 3E–J); correlated with poor DSS in ACC, ESCA, KIRC, KIPP, MESO and PAAD by 0.002, 0.036, 0.004, 0.010, 0.038 and 0.039, respectively (Figures 2K–P); correlated with poor DFI in ACC, LGG, LUAD, OV and PAAD by 0.026, 0.005, 0.004 m 0.046 and 0.001, respectively (Figures 2Q–U); and correlated with poor PFI in ACC, CESC, KIRC, KIPP, PAAD and SARC by 0.005, 0.028, 0.001, 0.007, 0.020 and 0.042, respectively (Figure 2 V-AA).

### 3.3 The relationships between *KRAS* expression levels with clinicopathological features

We explored the connection between the clinicopathological characteristics and various tumor stages according to *KRAS* expression levels in different pathology grades. These data suggest that high *KRAS* expression levels had effects at severe stages; correlation analysis showed in ACC, COAD, ESCA, KIRC, KIRP, LIHC, MESO, SKCM and STAD (Figures 3A–I). In addition, high *KRAS* expression levels showed a correlation with TMB in 14 tumors: BLCA, COAD, HNSC, LIHC, LUAD, LUSC, LUSC, PAAD, OV, PRAD, SARC, STAD, THYM, UCEC and UVM (Figure 3J); high *KRAS* expression levels showed a correlation with MSI in 8 tumors: BRCA, COAD, DLBC, READ, SKCM, STAD, TGCT and UCEC (Figure 3K).

### 3.4 The relationships between *KRAS* expression levels and tumor microenvironment

**FIGURE 4:**
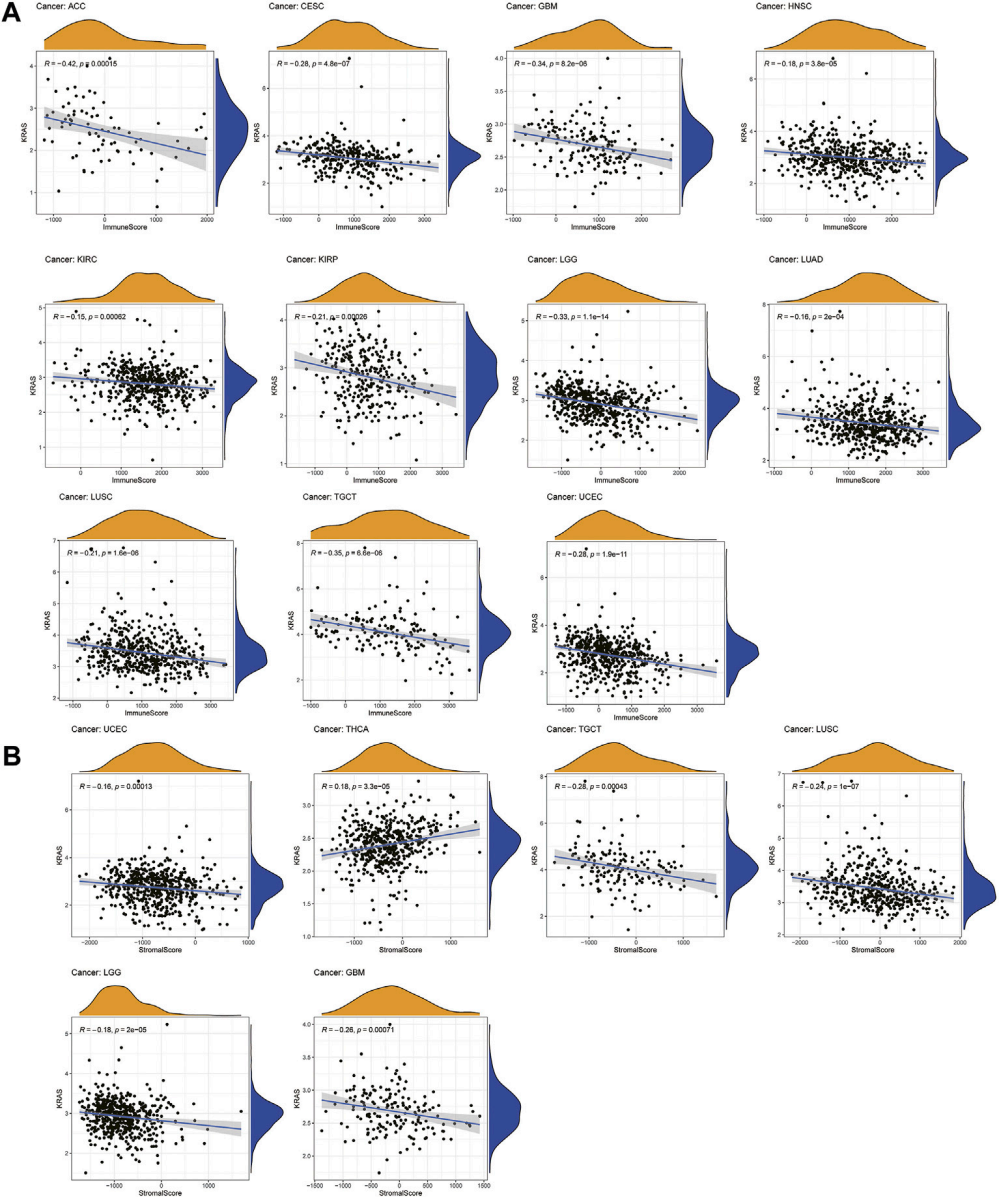
Relationships between *KRAS* expression and tumor microenvironment. **(A)** Immune score, **(B)** Stromal score.

A total of 22 immune-infiltrating cells were introduced to analyze the tumor microenvironment by 33 tumor expression data. Looking at Figure 4A, it is apparent that the immune scores of ACC, CESC, GBM, HNSC, KIRC, KIRP, LGG, LUAD, LUSC, TGCT and UCEC were negatively associated with *KRAS* expression; from Figure 4B above we can see that the stromal scores in THCA showed a causal negative relationship with *KRAS* expression levels, and GBM, LGG, LUSC, TGCT and UECE showed a causal negative relationship with *KRAS* expression levels.

### 3.5 The associations of *KRAS* expression levels with tumor immune cell infiltration levels

**FIGURE 5:**
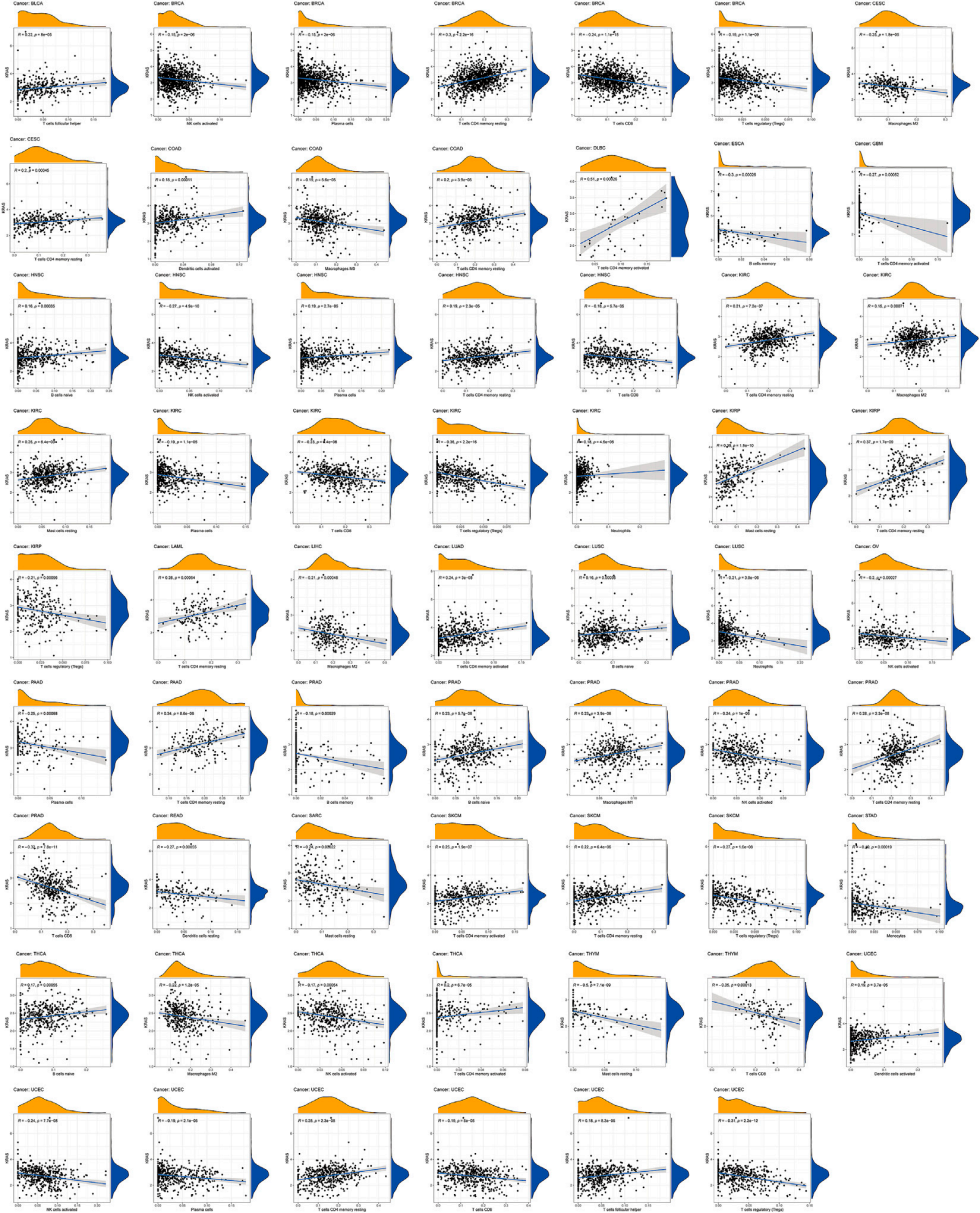
Relationships between *KRAS* expression and different types of immune cells infiltration level in tumors.

We established 22 cell expression datasets based on the immune-infiltrating levels to explore the potential effect of *KRAS* expression levels on the tumor immune cell infiltration. The results of the correlational analysis are shown in Figure 5; we could demonstrate that higher expression levels of *KRAS* were positively correlated with the infiltration levels of follicular helper T cells, activated memory CD4 T cells, activated dendritic cells, activated NK cells, naïve B cells, resting plasma cells, resting mast cells, resting CD4 T cells and follicular helper T cells, which were negatively correlated with the infiltration levels of activated NK cells, plasma cells, CD8 T cells, regulatory T cells (Tregs), M2 macrophages, M0 macrophages, memory B cells, activated memory T cells, memory CD4 T cells, activated NK cells, CD8 T cells, neutrophils, and monocytes in tumor cancers. High *KRAS* expression levels were positively correlated with the infiltration level of follicular helper T cells in BLCA. High *KRAS* expression levels were positively correlated with the infiltration level of resting CD4 T cells in BRCA. High *KRAS* expression levels were negatively correlated with the infiltration levels of activated NK cells, plasma cells, CD8 T cells and regulatory T cells (Tregs) in BRCA. High *KRAS* expression levels were positively correlated with the infiltration level of resting CD4 T cells in CESC. High *KRAS* expression levels were negatively correlated with the infiltration levels of M2 macrophages in CESC. High *KRAS* expression levels were positively correlated with the infiltration level of activated dendritic cells and resting CD4 T cells in COAD. High *KRAS* expression levels were negatively correlated with the infiltration levels of M0 macrophages in COAD. High *KRAS* expression levels were positively correlated with the infiltration level of activated memory CD4 T cells in DLBC. High *KRAS* expression levels were negatively correlated with the infiltration levels of memory B cells in ESCA. High *KRAS* expression levels were negatively correlated with the infiltration levels of activated memory CD4 T cells in GBM. High *KRAS* expression levels were positively correlated with the infiltration level of naïve B cells, plasma cells and memory CD4 T cells resting in HNSC. High *KRAS* expression levels were negatively correlated with the infiltration levels of activated NK cells and CD8 T cells in HNSC. High *KRAS* expression levels were positively correlated with the infiltration level of M2 macrophages, resting memory CD4 T cells, resting MAST cells and neutrophils in KIRC. High KRAS expression levels were negatively correlated with the infiltration levels of regulatory T cells (Tregs), CD8 T cells and plasma cells in KIRC. High KRAS expression levels were positively correlated with the infiltration level of resting mast cells and resting memory CD4 T cells in KIRP. High *KRAS* expression levels were negatively correlated with the infiltration levels of regulatory T cells (Tregs) in KIRP. High *KRAS* expression levels were positively correlated with the infiltration level of memory resting memory CD4 T cells in LAML. High KRAS expression levels were positively correlated with the infiltration levels of memory-activated CD4 T cells in LUAD. High KRAS expression levels were positively clinically relevant to the infiltration levels of memory B cells in LUSC. High KRAS expression levels were negatively clinically relevant to the infiltration levels of neutrophils in LUSC. High KRAS expression levels were negatively correlated with the infiltration levels of activated NK cells in OV. High *KRAS* expression levels were positively correlated with the infiltration levels of memory CD4 T cells resting in PAAD. High *KRAS* expression levels were negatively correlated with the infiltration levels of plasma cells in PAAD. High *KRAS* expression levels were positively correlated with the infiltration levels of naïve B cells, M1 macrophages and resting memory CD4 T cells in PRAD. High KRAS expression levels were negatively correlated with the infiltration levels of memory B cells, activated NK cells and CD8 T cells in PRAD. High KRAS expression levels were negatively correlated with the infiltration levels of dendritic cells resting in READ. High KRAS expression levels were negatively correlated with the infiltration levels of resting mast cells in SARC. High *KRAS* expression levels were positively correlated with the infiltration levels of activated memory CD4 T cells and resting CD4 T cells in SKCM. High *KRAS* expression levels were negatively correlated with the infiltration levels of regulatory T cells (Tregs) in SKCM. High *KRAS* expression levels were negatively correlated with the infiltration levels of Mococytes in STAD. High KRAS expression levels were positively correlated with the infiltration levels of naïve B cells and memory CD4 T cells activated in THCA. High *KRAS* expression levels were negatively correlated with the infiltration levels of M2 macrophages and NK cells activated in THCA. High *KRAS* expression levels were negatively correlated with the infiltration levels of resting mast cells and CD8 T cells in THYM. High KRAS expression levels were positively correlated with the infiltration levels of activated dendritic cells, resting memory CD4 T cells, and follicular helper T cells in UCEC. High *KRAS* expression levels were negatively correlated with the infiltration levels of activated NK cells, plasma cells, CD8 T cells and regulatory T cells (Tregs).

### 3.6 Functional enrichment analysis of KRAS

**FIGURE 6:**
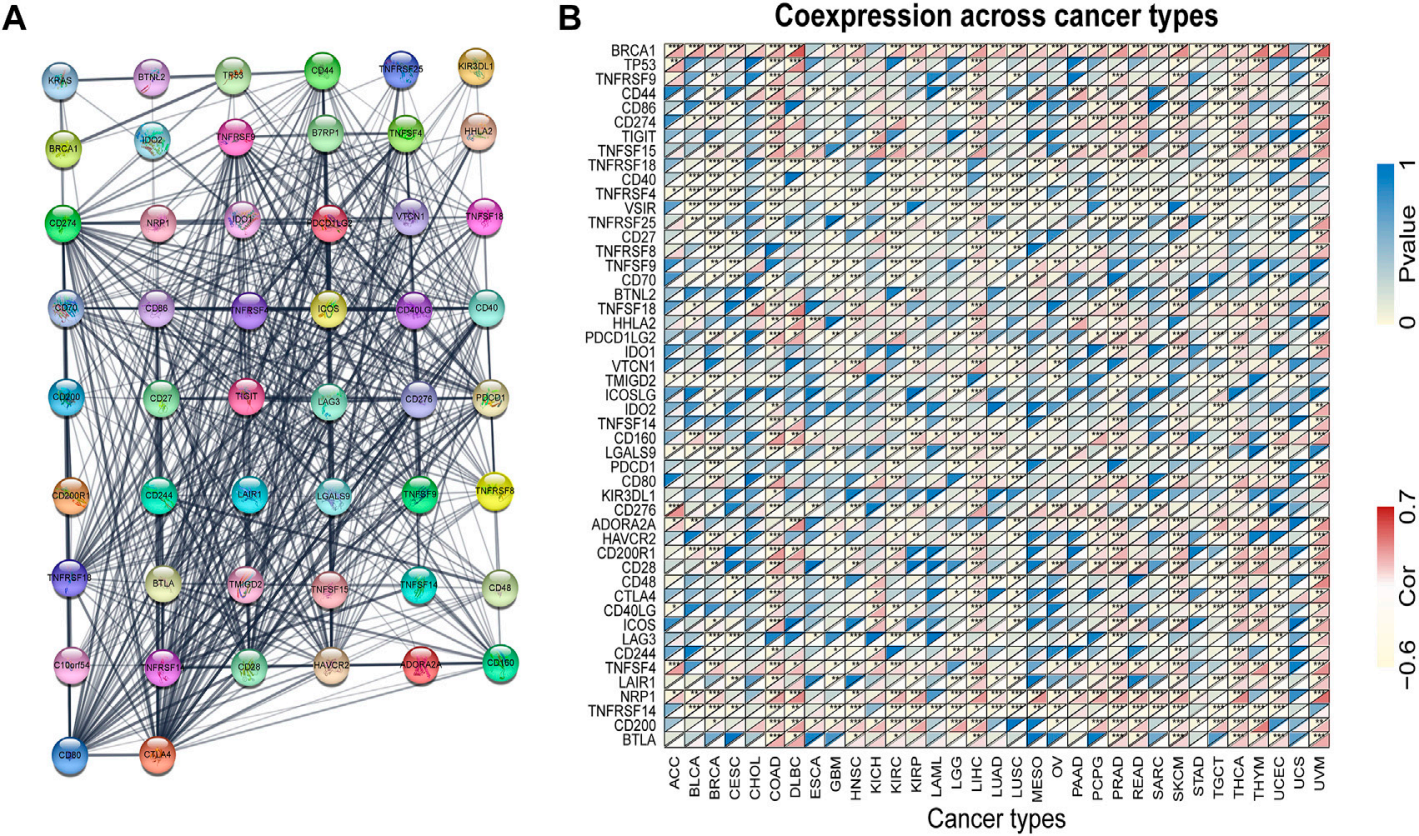
*KRAS* protein-protein network and expression relationships between *KRAS* and related genes. **(A)** PPI network for *KRAS*-interaction genes. **(B)** Correlation between *KRAS* expression and related genes (immune checkpoint genes and interacted related genes) expression.

*KRAS* is a signal transducer protein that binds to GTP in the MAPK pathway, and mutations have been found in a quarter of cancers. We fabricated the PPI network using the STRING database based on *KRAS* and *KRAS*-related genes (Figure 6A). Furthermore, we utilized the genes from the PPI network to analyze the association with *KRAS* expression. The results indicate that the great mass of immune checkpoint genes and *KRAS*-related genes were correlated with 33 kinds of tumor cancers (Figure 6B).

**FIGURE 7:**
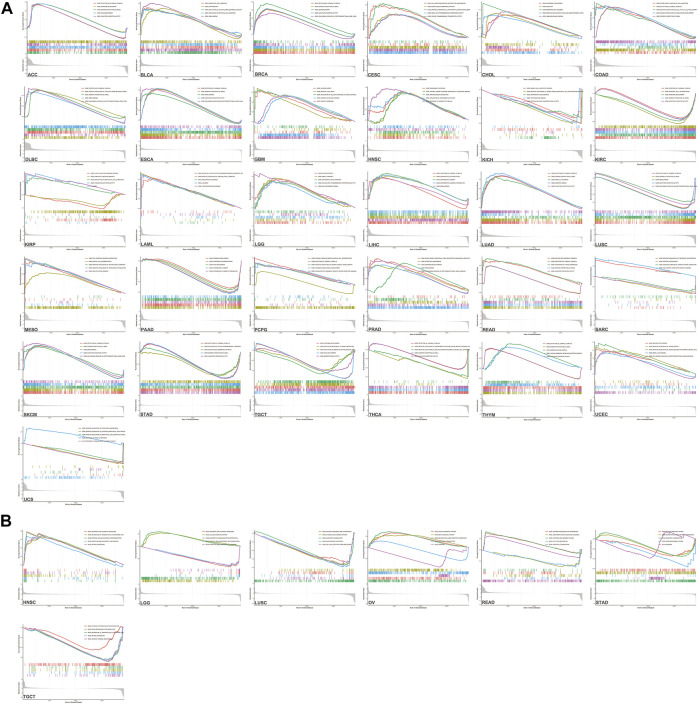
GSEA enrichment analysis of *KRAS* **(A)** Go enrichment analysis in various tumors. **(B)** KEGG enrichment analysis in various tumors.

We utilized GSEA to discuss the molecular circadian mechanism in multiple cancers. *KRAS* expression was involved in more than 96 kinds of GO (Gene Ontology) pathways in 33 tumor cancers (Figure 7A). In ACC, high expression levels of *KRAS* proteins were involved in epidermal development, neural signal response and sensory perception of a smell. In BLAC, high *KRAS* expression levels affect the cellular amide metabolic process, mRNA binding sites, cell migration and cell keratinization. Furthermore, high *KRAS* gene expression levels were related to the detection of chemical stimuli in COAD, DLBC, ESCA, LIHC, LUAD, LUSC, PCPG, READ, SKCM, STAD and THYM. *KRAS* proteins could be involved in mRNA binding in LUAD, UCEC, DLBC, ESCA, LIHC, LUSC, PRAD and SKCM. High *KRAS* expression levels were involved in recognizing and characterizing the olfactory stimulus signal in BRCA, COAD, DLBC, ESCA, LUAD, LUSC, READ, SKCM, STAD and THYM. On the other hand, KRAS is an essential member of keratinocyte differentiation in LIHC, OV and PAAD. The production of the immunoglobulin complex was due to the high *KRAS* expression in CHOL, PRAD, UVM, HNSC and UVM. Intermediate filament formation was related to high *KRAS* expression in CESC, LIHC, PAAD and OV. The result demonstrated that *KRAS* genes were involved in the formation of amyloid fibrils in LAML and MESO.

Interestingly, KEGG analysis promoted revealed the expression levels of *KRAS* genes involved in more than 20 kinds of pathways in 7 tumors. Increased *KRAS* expression is involved in the calcium signaling pathway in LUSC, OV, READ and STAD. In LUSC and READ, antigen processing and presentation seemed to have a correlation with high *KRAS* expression levels. Neuroactive ligand–receptor interactions are also effected in LGG, OV and STAD. High *KRAS* expression levels promoted ascorbate and alternate metabolism in HNSC and LGG (Figure 7B). There are many other GO ontology and KEGG pathways related to *KRAS* expression.

## 4 Discussion

The RAS gene family is one of the most widely studied families of cancer-related genes. Previous studies have shown that *KRAS* is the most common mutation of the three altered genes (Wennerberg et al., 2005). KRAS-related carcinogenic mutations are prevalent in human cancer, occurring in 17%–25% of all cancers. The results indicated that cancer could lead to the abnormal expression of *KRAS* in tumor tissues. In addition, we analyzed the *KRAS* expression levels on different pathology grades; high expression levels of *KRAS* genes affected the degree of tumor deterioration at serious stages, and correlation analysis showed in ACC, COAD, ESCA, KIRC, KIRP, LIHC, MESO, SKCM and STAD. Many studies have shown that *KRAS* has an essential relationship with the occurrence of cancer (Roberts and Stinchcombe, 2013). Therefore, *KRAS* has the potential to be introduced as a prognostic factor in many tumors.

TMB was calculated by the number of noninherited mutations, which are considered to be an important genetic feature to evaluate tumor tissue (Merino et al., 2020). As a genomic biomarker that predicts a good response to an immune checkpoint inhibitor (Kim et al., 2019). MSI is generated from impaired DNA mismatch repair, which occurs during gene duplication (Buecher et al., 2013). MSI p roduction is not random, and different target genes will lead to different phenotypes and pathologies and affect the pathogenesis of many kinds of cancer (Imai and Yamamoto, 2008). In addition, high *KRAS* expression levels showed a correlation with TMB in 14 tumors: BLCA, COAD, HNSC, LIHC, LUAD, LUSC, LUSC, PAAD, OV, PRAD, SARC, STAD, THYM, UCEC and UVM; with MSI in BRCA, COAD, DLBC, READ, SKCM, STAD, TGCT and UCEC. TMB and MSI are relatively new biomarkers, and there is still a need to perform more studies.

RAS mutations participating in the production of human cancer cells have been studied in many types of research. Nevertheless, its potential mechanism and molecular regulatory mechanism need to be further clarified (Hu et al., 2022b). *KRAS* mutation is considered to be one of the most common genome variation in non-small cell lung cancer and is associated with a clinical background and pathological features (Suda et al., 2010). The pro-tumor inflammation caused by *KRAS* is associated with immune regulation, which leads to immune escape in the TME (Hamarsheh et al., 2020). The immune and stromal scores reflected the proportion of cancer cells in tumor tissue; we established 22 kinds of expression datasets by immune-infiltrating cells. Our research suggested that *KRAS* was associated with immunotherapeutic effects and cell viability in multiple tumors and had excellent potential as a cancer-targeted drug.

*KRAS* has been reported as a biomarker in multiple cancers (Petrelli et al., 2013; Siddiqui and Piperdi, 2010; Yang et al., 2013). In our study, the KRAS expression levels in various tumor cells were associated with prognosis and immune cell infiltration. From the GO ontology and KEGG pathways, the expression of *KRAS* plays an essential role in different stages of cancers with multiple functions. We found that *KRAS* interacts with several cancer-related genes from the PPI network, but their mechanism needs further study. Based on our study, *KRAS* may be a reliable prognostic biomarker for cancer patients in the course of diagnosis and treatment.

## 5 Conclusion

We studied the novel cancer-related gene *KRAS*, which belongs to the *RAS* gene family, in 33 kinds of tumors. This study has shown that *KRAS* were significantly different in 12 tumor tissues compared to normal tissues. Furthermore, the second significant finding was that the prognostic value of OS, DSS and DFI and DSS correlated with KRAS expression levels in 4, 3, 4 and 6 kinds of tumors, separately. In addition, *KRAS* expression levels were associated with tumor mutation burden (TMB) and microsatellite instability (MSI) in 14 and 8 tumors. As for 22 immune infiltrating cells, immune and stromal scores showed 11 and 6 kinds of tumors correlated with *KRAS* expression levels based on the tumor purity. The PPI network and functional enrichment participate in different biological metabolic pathways. Go and KEGG enrichment analysis revealed that *KRAS* was connected with more than 96 GO pathways in 33 tumor cancer cells and more than 20 kinds of KEGG pathways in 7 tumors, indicated that KRAS expression was involved in epidermal development, neural signal response, sensory perception of a smell, metabolic process, mRNA binding sites, cell migration and cell keratinization in multiple tumors. Our study suggested that *KRAS* may be a promising prognostic biomarker for cancer diagnosis and treatment.

## Data Availability

The original contributions presented in the study are included in the article/Supplementary Material, further inquiries can be directed to the corresponding author.
